# Genome-Wide Identification and Functional Analysis of *Salvia miltiorrhiza* MicroRNAs Reveal the Negative Regulatory Role of Smi-miR159a in Phenolic Acid Biosynthesis

**DOI:** 10.3390/ijms25105148

**Published:** 2024-05-09

**Authors:** Hong Zhou, Maochang Jiang, Jiang Li, Yayun Xu, Caili Li, Shanfa Lu

**Affiliations:** 1 State Key Laboratory for Quality Ensurance and Sustainable Use of Dao-di Herbs, Institute of Medicinal Plant Development, Chinese Academy of Medical Sciences & Peking Union Medical College, Beijing 100193, China; zhouhong1013@126.com (H.Z.); 17852320203@163.com (M.J.); jli@implad.ac.cn (J.L.); 14418120@njau.edu.cn (Y.X.); 2 Key Lab of Chinese Medicine Resources Conservation, State Administration of Traditional Chinese Medicine of the People’s Republic of China, Institute of Medicinal Plant Development, Chinese Academy of Medical Sciences & Peking Union Medical College, Beijing 100193, China; 3 College of Pharmacy, Shenzhen Technology University, Shenzhen 518118, China

**Keywords:** non-coding RNA, rosmarinic acid, salvianolic acid B, smi-miR159a, traditional Chinese medicine

## Abstract

MicroRNAs (miRNAs) are a group of endogenous small non-coding RNAs in plants. They play critical functions in various biological processes during plant growth and development. *Salvia miltiorrhiza* is a well-known traditional Chinese medicinal plant with significant medicinal, economic, and academic values. In order to elucidate the role of miRNAs in *S. miltiorrhiza*, six small RNA libraries from mature roots, young roots, stems, mature leaves, young leaves and flowers of *S. miltiorrhiza* and one degradome library from mixed tissues were constructed. A total of 184 miRNA precursors, generating 137 known and 49 novel miRNAs, were genome-widely identified. The identified miRNAs were predicted to play diversified regulatory roles in plants through regulating 891 genes. qRT-PCR and 5′ RLM-RACE assays validated the negative regulatory role of smi-miR159a in *SmMYB62*, *SmMYB78*, and *SmMYB80*. To elucidate the function of smi-miR159a in bioactive compound biosynthesis, smi-miR159a transgenic hairy roots were generated and analyzed. The results showed that overexpression of smi-miR159a caused a significant decrease in rosmarinic acid and salvianolic acid B contents. qRT-PCR analysis showed that the targets of smi-miR159a, including *SmMYB62*, *SmMYB78,* and *SmMYB80*, were significantly down-regulated, accompanied by the down-regulation of *SmPAL1*, *SmC4H1*, *Sm4CL1*, *SmTAT1*, *SmTAT3*, *SmHPPR1*, *SmRAS,* and *SmCYP98A14* genes involved in phenolic acid biosynthesis. It suggests that smi-miR159a is a significant negative regulator of phenolic acid biosynthesis in *S. miltiorrhiza*.

## 1. Introduction

Plant small RNAs (sRNAs) are crucial regulators of gene expression at both transcriptional and post-transcriptional levels [1,2]. They could be classified into two major groups, including microRNAs (miRNAs) and small interfering RNAs (siRNAs), based on their biogenesis pathways and functions [3]. MiRNAs originate from single-stranded primary miRNA transcripts (pri-miRNAs), which form imperfect stem-loop structures and are cleaved by a dicer-like 1 (DCL1)-containing protein complex in the nucleus. It results in the generation of a hairpin structure with approximately 60–300 nucleotides in length, namely miRNA precursor (pre-miRNA). Further process of pre-miRNAs generates a miRNA/miRNA* duplex, which is then unwound into single-stranded mature miRNA and miRNA*. The mature miRNA can be incorporated into an RNA-induced silencing complex (RISC) to repress gene expression, while the generated miRNA* is usually degraded in the cytoplasm [4].

To date, deep sequencing technology has led to the identification of a large number of miRNAs in various organisms. The latest release of miRBase (version 22.0) includes 38,589 miRNA loci from 271 species, resulting in 48,885 mature miRNA products. As the second most abundant sRNAs in plants, miRNAs play a crucial role as endogenous regulators in various biological processes, such as plant development, secondary metabolism, environmental stress response, and disease resistance. Like other gene regulatory systems, miRNAs show both diversity and conservation across plant species. Some miRNAs are deeply conserved across different plant species, such as miR156/157 that controls flowering time through the regulation of *SQUAMOSA PROMOTER BINDING-LIKE* (*SPL*) transcription factors [5,6,7]; miR159 that targets *GAMYB* gene transcripts to regulate *Arabidopsis* programmed cell death (PCD) [8], flowering [9], strawberry fruit development [10], grape flower development [11], and tomato fruit morphology [12]; miR160 and miR167 that target *AUXIN RESPONSE FACTOR* (*ARF*) transcripts to regulate root development [13]; and miR172 that regulates flower development through regulating *APETALA2* (*AP2*) transcription factor genes [14,15]. However, the majority of miRNAs are specific to certain plant families or species. MiRNAs or miRNA families newly emerged during plant evolution possess unique properties and functions [16]. Evolutionary trajectory and functional diversity highlight the importance to comprehensively identify miRNAs in a plant species.

Bioactive compounds, usually secondary metabolites, are important to human beings. More and more evidence show that miRNAs play a crucial role in the biosynthesis of secondary metabolites in plants. For instance, *Arabidopsis* miR156 may cleave the transcripts of the *SPL9* gene, which in turn regulates the expression of the favanone-3-hydroxylase gene (*F3H*), anthocyanidin synthase gene (*ANS*), and dihydrofavonol-4-reductase gene (*DFR*), subsequently affecting the accumulation of anthocyanins [17]. Grape miR828 and miR858 regulate the expression of MYB transcription factor gene *VvMYB114*, leading to enhanced contents of anthocyanins and flavonols [18]. Overexpression of *Arabidopsis* miR858a down-regulated the expression of several MYB transcription factor genes involved in flavonoid biosynthesis [19]. Additionally, ptr-miR397a plays a regulatory role in lignin biosynthesis through altering laccase gene expression in *Populus* [20].

*Salvia miltiorrhiza* Bunge, a member of the Lamiaceae family, is a well-known traditional Chinese medicinal plant. It has been used for centuries in Asia to treat cardiovascular and cerebrovascular diseases and is now recognized as a health supplement in Western countries as well [21,22]. Tanshinones and phenolic acids are two major classes of bioactive compounds produced in *S. miltiorrhiza*. Their biosynthesis and regulation have been intensively studied [23,24,25,26,27,28,29,30,31]. Recent results imply the importance of miRNAs in *S. miltiorrhiza*. For instance, smi-miR12112, a lineage-specific miRNA family, could be involved in phenolic compound metabolism through regulating polyphenol oxidase genes (*SmPPOs*) [32]. Smi-miR399 could be involved in secondary metabolism through regulating alkaline/neutral invertase genes (*SmNINVs*) [33]. Overexpression of smi-miR396b in *S. miltiorrhiza* hairy roots led to the repression of salvianolic acid biosynthesis and to the enhancement of tanshinone production through regulating the expression of *SmGRF*, *SmHDT1*, and *SmMYB37/4* genes [34]. Transgenic *S. miltiorrhiza* lacking smi-miR408 showed increased levels of rosmarinic acid (RA) and salvianolic acid B (SalB) [35]. Overexpression of smi-miR858a caused a significant reduction in tanshinones and phenolic acids, two major classes of bioactive compounds in *S. miltiorrhiza* [36]. 

High-throughput miRNA identification was conducted for *S. miltiorrhiza*. A total of 492 miRNAs and 69 targets for 25 miRNAs were firstly reported in 2014, when there was no *S. miltiorrhiza* whole-genome sequence available [37]. The authors mapped sRNAs to miRNA precursors from other plant species for *S. miltiorrhiza* miRNA identification [37]. This is not a common method in miRNA analysis, indicating that the results obtained require further validation. In 2016, Zhang et al. reported the sequencing of two small RNA libraries from roots of the first- and the second-year *S. miltiorrhiza* plants [38]. The authors mapped sRNAs to the transcriptome data of *S. miltiorrhiza* for miRNA identification. It resulted in the identification of 117 *S. miltiorrhiza* miRNA candidates [38]. Recently, four *S. miltiorrhiza* genomes have been sequenced and assembled [39,40,41,42]. It provides an opportunity for the genome-wide identification of *S. miltiorrhiza* miRNAs.

With the aim to identify *S. miltiorrhiza* miRNAs genome-widely and test whether smi-miR159a are involved in regulating the biosynthesis of bioactive compounds, we constructed and sequenced six sRNA libraries and a degradome library. Genome-wide analyses identified a total of 184 miRNA loci, representing 186 miRNAs. A total of 891 miRNA targets were also identified, of which *SmMYB62*, *SmMYB78,* and *SmMYB80* were targeted by smi-miR159a. Overexpression of smi-miR159a in *S. miltiorrhiza* hairy roots caused a significant decrease in SalB and RA, suggesting the regulatory roles of smi-miR159a in phenolic acid biosynthesis in *S. miltiorrhiza*. To the best of our knowledge, this is the first report on the involvement of miR159 in regulating active compound biosynthesis in medicinal plants.

## 2. Results and Discussion

### 2.1. High-Throughput Sequencing of S. miltiorrhiza sRNAs

**Figure 1:**
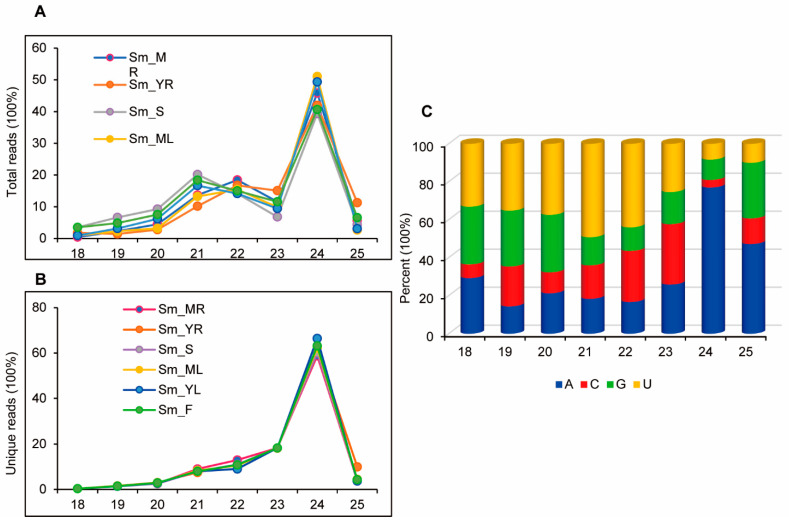
Sequencing analysis of small RNAs from mature roots (MR), young roots (YR), stems (S), mature leaves (ML), young leaves (YL) and flowers (F) of *S. miltiorrhiza*. (**A**) Length distribution of total sRNAs in each tissue. (**B**) Length distribution of unique sRNAs in each tissue. (**C**) Percentage of the first base of 18–25 nt in unique sRNAs.

For the genome-wide identification of *S. miltiorrhiza* sRNAs, six sRNA libraries from mature roots, young roots, stems, mature leaves, young leaves, and flowers were constructed. Deep sequencing of the libraries constructed yielded approximately 70 million clean reads (Table S1). The length distribution patterns of sRNAs from each library were similar. The majority (63%) of the sRNAs across all libraries analyzed were 24 nt long, indicating the abundance of endogenous siRNAs (Figure 1A). The proportions of 21, 22, and 23 nt sRNAs were 8.4%, 10.8%, and 18.2% of total sRNAs, respectively (Figure 1A,B). Overall, more than 95% of the sRNAs fell within the range of 21–24 nt (Figure 1A,B), which is consistent with the cleavage products of DCLs [43,44,45]. The number of unique sRNAs ranged from 2.8 to 6.2 million across all libraries analyzed. Among them, 24 nt small RNAs were the most abundant (40.66%), followed by 21 (18.4%), 22 (15.4%), and 23 nt (11.5%). It indicates a normal distribution of sRNA lengths in comparison to the sRNAs in other plant species [46,47,48]. Analysis of the first nucleotide bias in each size group revealed that the 21 and 22 nt groups were enriched in sequences starting with uracil (U), while the 24 nt group had the highest percentage of sRNAs starting with adenine (A) compared with other size classes (Figure 1C). The findings are consistent with previous studies on *S. miltiorrhiza* [37,38] as well as other plant species, such as *Arabidopsis*, *Populus*, and *Panax ginseng* [46,47,48].

### 2.2. Identification of Known and Novel miRNAs in S. miltiorrhiza

In order to identify miRNAs in *S. miltiorrhiza*, unique sRNA sequences with at least two reads were mapped to the genome assembly of *S. miltiorrhiza* and predicted for precursor sequences using the psRobot software package v1.2 with the default parameters [49]. Precursor sequences with stem-loop structure as the minimal free energy folding form and the corresponding high reads of query smRNA were selected. After manual checking, a total of 184 precursor sequences generating 186 miRNAs were identified. The identified mature miRNA sequences were then aligned with known mature plant miRNAs in miRBase 22.0 using the BLAST program. A maximum of three mismatches was allowed. It resulted in the identification of 137 known miRNAs, belonging to 35 miRNA families (Table S2). Among them, the *MIR156*, *MIR166*, *MIR169,* and *MIR319* families had 10 members, followed by the *MIR399* family that had nine members. The remaining 49 miRNAs, derived from 49 precursors and having star sequences (miRNA*), were considered to be novel or *S. miltiorrhiza*-specific miRNAs (Table S2 and Figure S1). Most of the novel miRNAs were 21 nt long and had uracil (U) as the first nucleotide. 

In a previous study, Xu et al. reported the identification of 492 *S. miltiorrhiza* miRNAs through the alignment of *S. miltiorrhiza* sRNAs with miRNA precursors from other species [37]. Many of the reported miRNAs were highly homologous or shortened from the longer ones. It indicates that some of them could be derived from the same precursors. We checked these miRNAs through mapping them to the assembly of *S. miltiorrhiza* genome using psRobot software v1.2 [49]. Subsequent analysis identified 89 miRNA precursors (Table S3). Sequence comparison showed that all of them had been included in the 184 miRNA precursors we identified. 

In another previous study, Zhang et al. performed high-throughput sequencing of two small RNA libraries constructed from the roots of first-year and second-year *S. miltiorrhiza* plants. A total of 117 miRNAs were identified through mapping the sRNAs to *S. miltiorrhiza* transcriptome data [38]. We mapped these miRNAs to the assembly of *S. miltiorrhiza* genome. It resulted in the identification of 84 miRNA precursors (Table S4), all of which had been included in the 184 miRNA precursors we identified, and 70 of which were included in the 89 miRNA precursors shown in Table S3.

### 2.3. Expression Patterns of S. miltiorrhiza miRNAs

**Figure 2:**
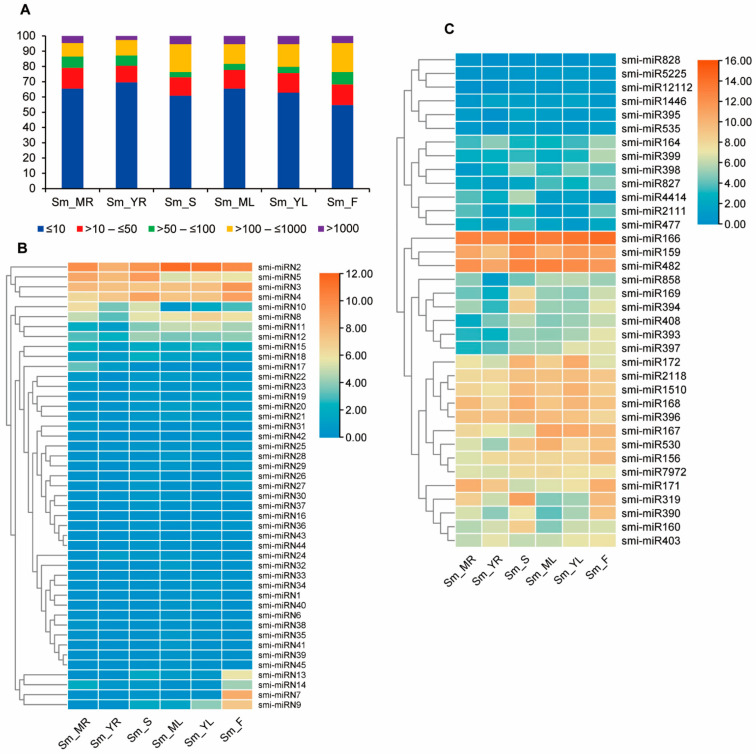
Expression patterns of miRNAs in mature roots (MR), young roots (YR), stems (S), mature leaves (ML), young leaves (YL) and flowers (F) of *S. miltiorrhiza*. (**A**) Percentage of miRNAs with different expression abundances in tissues analyzed. MiRNAs with normalized expression values less than 10, greater than 10 to 50, greater than 50 to 100, greater than 100 to 1000, and greater than 1000 were classified into very lowly, lowly, moderately, highly, and very highly expressed, respectively. (**B**,**C**) Heatmaps of expression levels of novel (**B**) and known (**C**) miRNAs. The color scale represents log_2_ (transformed normalized expression values). The heatmap was created using TBtools [51].

In order to gain preliminary knowledge on the function of *S. miltiorrhiza* miRNAs, expression patterns of the 186 miRNAs identified were analyzed in four tissues of *S. miltiorrhiza*. The expression level (read count) of each miRNA in a tissue sample was adjusted to reads per million (RPM) by applying a scaling factor through DESeq2 [50]. The results showed substantial expression differences among *S. miltiorrhiza* miRNAs. Based on normalized expression values, we classified miRNA expression levels into five distinct categories, including extremely low, low, moderate, high, and extremely high (Figure 2A). Among them, very low expression accounted for the largest proportion, representing 54–69% of miRNAs. The high or very high levels accounted for 12–23% of miRNAs (Figure 2A). Generally, known miRNA families were ubiquitously expressed at high levels across tissues. For instance, the *MIR166* family had the highest abundance in *S. miltiorrhiza* and was highly expressed in flowers. It had a total of 76,159 RPM, accounting for 44.8% of total known miRNA reads. The other highly expressed miRNA families include *MIR482*, *MIR159*, and *MIR164*. Except them, other known miRNA families exhibited less abundant. Each of them had less than 5000 RPM (Table S5). Among them, the *MIR1446*, *MIR395*, *MIR535*, *MIR5225*, *MIR12112,* and *MIR828* family had less than 10 total RPM (Table S5). In addition, the expression level of miRNAs varied among tissues. Of the 137 miRNAs, three showed predominant expression in leaves, fourteen in flowers, six in stems, and two in roots. The other 112 were expressed relatively high in at least two tissues analyzed (Figure 2B,C). For instance, the *MIR167* family was expressed with more than 1900 RPM in mature leaves, but less than 90 RPM in stems (Table S5). Smi-miR828 and smi-miR12112 were not detected in young roots.

**Figure 3:**
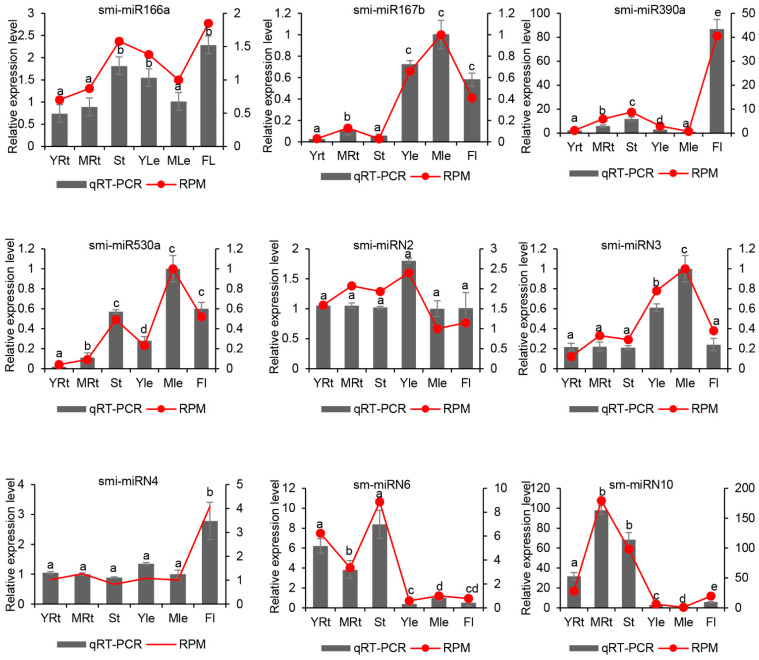
qRT-PCR validation of miRNA expression obtained through sRNAome analysis. The expression of each miRNA in different tissues was normalized with the expression of 5.8S rRNA. Data are the means ± SD from three biological replicates. Letters a–e indicate significantly different at *p* ≤ 0.05 in different tissues.

Compared with known miRNAs, the expression levels of novel miRNAs were generally low. Except smi-miRN2 that yielded more than 8000 RPM, the levels of other novel miRNAs were all below 2000 RPM, and 32 of the 49 novel miRNAs had less than 10 RPM (Table S6). To validate the expression patterns, we further selected four known and four novel miRNAs for qRT-PCR analysis (Figure 3). The results showed that, although the patterns of smi-miRN2 and smi-miRN4 in some tissues were different from those obtained through sRNAome analysis, the general miRNA expression trends obtained through qRT-PCR detection and sRNAome analysis were consistent.

### 2.4. Identification of miRNA Targets in S. miltiorrhiza

MiRNAs play regulatory roles in plants mainly through cleaving target transcripts. Therefore, prediction and verification of miRNA targets are fundamental for understanding miRNA functions. Plant miRNAs show perfect or near-perfect sequence complementarity to their targets and mainly cleave targets in a site corresponding to the 10th miRNA nucleotide from the 5′ end [52]. This feature ensures an effective prediction of miRNAs targets through bioinformatic analysis. Here, the targets of all identified miRNAs were predicted using the computational algorithm, psRNATarget, with the penalty score threshold of 0–3.0 [53]. It resulted in the prediction of 662 targets for known miRNAs and 229 for novel miRNAs (Tables S7 and S8). The number of targets varied among miRNA families, ranging from 2 to 57 for each miRNA family. No targets were predicted for smi-miR398b, smi-miR403, and smi-miR5225. It could be due to the incompleteness of *S. miltiorrhiza* genome assembly or the stringency of penalty score cut-off threshold. 

In order to validate the targets, we constructed and sequenced a degradome library from the aforementioned six tissues of *S. miltiorrhiza*. A total of 10,188,836 clean reads were obtained. An analysis of degradome sequences, 186 miRNAs and 891 predicted targets using the CleaveLand pipeline [54] confirmed that 188 predicted targets were indeed cleaved by miRNAs (Tables S7 and S8). The validated targets were classified into four classes (0, 1, 2, and 4) by the pipeline based on the abundance of the diagnostic cleavage tag relative to the overall profile of degradome tags [54]. The corresponding target number for each of the class 0, 1, 2, and 4 was 17, 17, 13, and 141, respectively.

**Figure 4:**
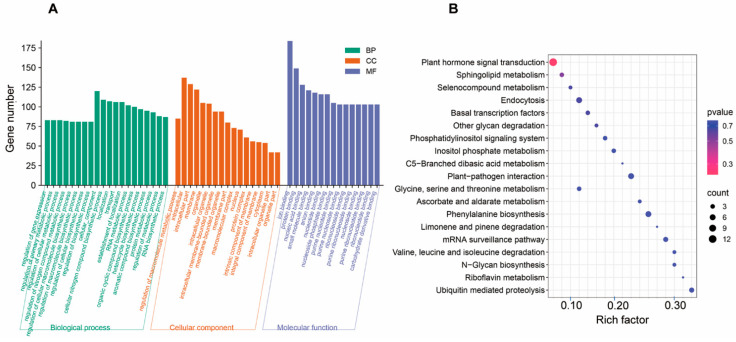
Gene Ontology (GO) and KEGG annotation of predicted targets. (**A**) Function classifications of GO terms of genes targeted by differentially expressed miRNAs. X-axis corresponds to Go classification. Y-axis indicates gene number of each term within Go category. (**B**) KEGG enrichment analysis of genes targeted by differentially expressed miRNAs. X-axis indicates the names of KEGG metabolic pathways. Y-axis indicates the Rich factor.

In order to further explore the function of miRNAs, predicted targets were analyzed using Gene Ontology (GO) functional classification and pathway enrichment from the Kyoto Encyclopedia of Genes and Genomes (KEGGs). The results showed significant enrichment of various metabolic processes, such as phenylpropanoid metabolism and cyclic compound biosynthetic processes. In addition, various transcriptional processes were also enriched, such as the regulation of gene expression and biosynthetic processes (Figure 4A). The most enriched KEGG pathways were plant hormone signal transduction, transcription factors, and phenylalanine biosynthesis. It suggests the importance of miRNAs and their targets in *S. miltiorrhiza* (Figure 4B).

Some targets of known *S. miltiorrhiza* miRNAs were homologous to previously identified miRNA targets, which is consistent with previous results showing the conserved regulatory roles of some plant miRNAs, such as the regulation of miR156/157 on *SPL* transcription factor genes [5,7], the regulation of miR159, miR319, miR828, and miR858 on *MYB* transcription factor genes [55,56], and the regulation of miR168 and miR403 on *AGO1* and *AGO2* genes, respectively [57]. Transcription factor genes made up the majority of such miRNA targets [43,44,45]. Consistently, a total of 124 transcription factor genes, such as *SmMYBs*, *SmARFs*, and *SmNACs*, were predicted to be targets of known *S. miltiorrhiza* miRNAs.

In addition, targets were also identified for 44 novel *S. miltiorrhiza* miRNAs. Unlike known miRNAs, most of the novel miRNAs had few targets (Tables S7 and S8). Functional annotation of these targets indicated that novel miRNAs could also be involved in signaling pathways and biological and metabolic processes. For example, smi-miRN2 could be involved in genome organization and maintenance through targeting structural maintenance of chromosomes protein gene. smi-miRN11 could be involved in signaling pathways through targeting tetratricopeptide repeat gene. smi-miRN39 could be involved in the glycolytic pathway through targeting glyceraldehyde-3-phosphate dehydrogenase (GAPDH) gene. smi-miRN9 could be involved in the methylation process through affecting the expression of histone-lysine *N*-methyltransferase suvr2-like gene.

### 2.5. Expression Analysis and 5′ RLM-RACE Validation of Smi-miR159 Targets

**Figure 5:**
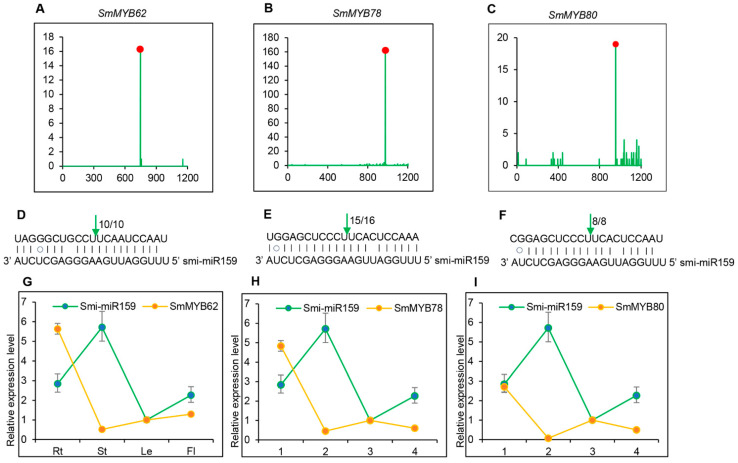
Smi-miR159a targets *SmMYBs* for cleavage. (**A**–**C**) Validation of Smi-miR159a-directed cleavage of *SmMYB62*, *SmMYB78,* and *SmMYB80* using degradome data. X-axis shows nucleotide (nt) positions of targets. Y-axis shows the reads obtained through degradome sequencing. Red spots indicate the products resulted from Smi-miR159a-directed cleavage. (**D**–**F**) 5′ RLM-RACE validation of Smi-miR159a-directed cleavage. Vertical arrows indicate the cleavage sites of Smi-miR159a. The number alongside the arrows shows the frequency of clones. (**G**–**I**) qRT-PCR analysis of smi-miR159a, *SmMYB62*, *SmMYB78*, and *SmMYB80*. Relative expression of Smi-miR159a, *SmMYB62*, *SmMYB78*, and *SmMYB80* were quantified in total RNA isolated from roots (Rt), stems (St), leaves (Le), and flowers (Fl) by qRT-PCR. Transcript levels in leaves were arbitrarily set to 1 and the levels in other tissues were given relative to this. Error bars represent standard deviations of mean value from three biological and three technical replicates.

Previous studies showed that miR159 played significant roles in PCD, flowering, fruit development, lignin metabolism, and so on, through cleaving *GAMYB* gene transcripts [8,9,10,11,12]. Its regulatory role in bioactive compound biosynthesis has not been analyzed. In *S. miltiorrhiza*, there are two *MIR159* precursors that generate identical mature miRNA sequences, smi-miR159a and smi-miR159b, respectively. Computational analysis predicted that transcripts of five *SmMYBs*, including *SmMYB62*, *SmMYB78*, *SmMYB80*, *SmMYB96*, and *SmMYB99*, could be cleaved by smi-miR159s. To validate the prediction, *S. miltiorrhiza* degradome sequencing data were analyzed for these *SmMYBs*. The results showed that three of the five *SmMYBs*, including *SmMYB62*, *SmMYB78*, and *SmMYB80*, were indeed cleaved by smi-miR159s (Figure 5A–C). Rapid amplification of the 5′ complementary DNA ends (5′-RACE) further confirmed that the three *SmMYBs* were cleaved by smi-miR159s in vivo (Figure 5D–F). To verify the expression relationship between smi-miR159s and *SmMYBs*, qRT-PCR was carried out. The results showed that *SmMYB62*, *SmMYB78*, and *SmMYB80* exhibited opposite expression trends against smi-miR159s (Figure 5G–I). Since the mature sequences of smi-miR159a and smi-miR159b were identical, we selected *smi-MIR159a* for subsequent functional studies.

### 2.6. Smi-miR159a Regulates the Biosynthesis of RA and SalB but Not Tanshinones in S. miltiorrhiza

**Figure 6:**
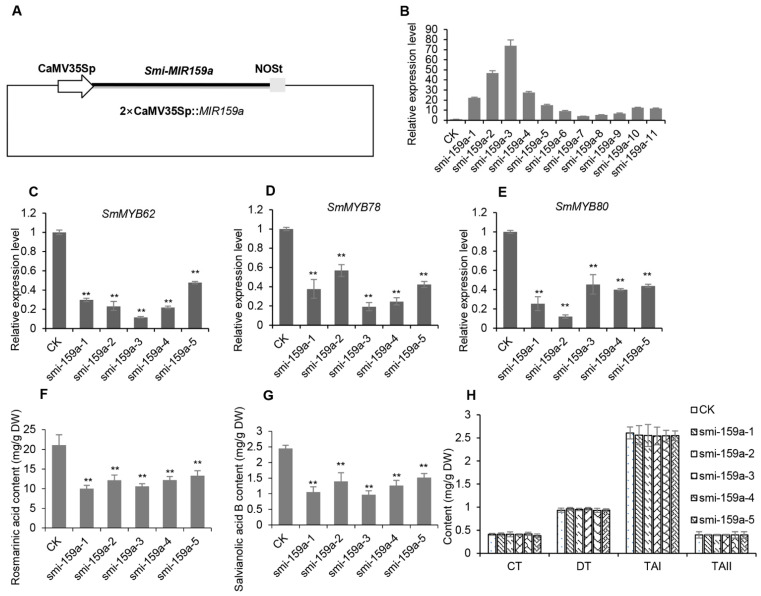
Overexpression of Smi-miR159a decreased the contents of RA and SalB but not tanshinones in *S. miltiorrhiza* hairy roots. (**A**) The 2×CaMV35Sp::*MIR159a* vector used for overexpression of smi-miR159a in *S. miltiorrhiza*. (**B**) Smi-miR159a levels in the control and smi-miR159a overexpression transgenic lines. (**C**–**E**) Relative expression levels of *SmMYB62*, *SmMYB7,* and *SmMYB80* in hairy roots of the control and smi-miR159a overexpression transgenics. The expression levels were analyzed by qRT-PCR. *SmUBQ10* was used as a control. Expression levels in the control (CK) were set to 1. Data are means ± SD of three biological replicates. (**F**,**G**) UPLC analysis of RA (**F**) and SalB (**G**) contents. (**H**) UPLC analysis of tanshinone contents. All data show mean ± SD of three biological replicates. Significant differences in comparison with the control were assessed with the Dunnett’s multiple comparison test (** *p* < 0.01).

To further elucidate the function of *smi-miR159a* in *S. miltiorrhiza*, a smi-miR159a overexpression vector, namely 2×CaMV35Sp::*MIR159a*, was constructed through inserting an 876 bp sequence containing a *smi-MIR159a* precursor into pCAMBIA1391 that contained a double 35S promoter (Figure 6A). The empty vector pCAMBIA1391 was used as the control. The vectors were then introduced into *Agrobacterium rhizogenes* ACCC10060, respectively. Genetic transformation of *S. miltiorrhiza* generated a total of 11 lines of hairy roots for 2×CaMV35Sp::*MIR159a* and three lines for pCAMBIA1391. qRT-PCR analysis showed that the levels of smi-miR159a in overexpression lines was 4–73 times of those in control (Figure 6B). On the contrary, the targets of smi-miR159a, including *SmMYB62*, *SmMYB78,* and *SmMYB80*, were significantly down-regulated (Figure 6C–E).

UPLC analysis of RA and SalB contents in transgenic hairy roots of the transgenic lines showed that RA was 21.09 mg/g DW in the control. It was reduced to 10.02 mg/g DW, 12.16 mg/g DW, 10.58 mg/g DW, 12.18 mg/g DW, and 13.30 mg/g DW in smi-miR159a overexpression lines smi-159a-1, smi-159a-2, smi-159a-3, smi-159a-4, and smi-159a-5, respectively (Figure 6F). Similarly, SalB was decreased to 1.06 mg/g DW, 1.39 mg/g DW, 0.97 mg/g DW, 1.26 mg/g DW, and 1.52 mg/g DW in lines smi-159a-1, smi-159a-2, smi-159a-3, smi-159a-4, and smi-159a-5, respectively, in comparison with the content of 2.46 mg/g DW in the control (Figure 6G). The results suggested that overexpression of smi-miR159a suppressed RA and SalB biosynthesis in *S. miltiorrhiza* hairy roots. UPLC analysis of tanshinones showed that the contents were not significantly altered in smi-miR159a overexpression hairy roots (Figure 6H). It indicated that smi-miR159a was not involved in the regulation of tanshinone biosynthesis. 

**Figure 7:**
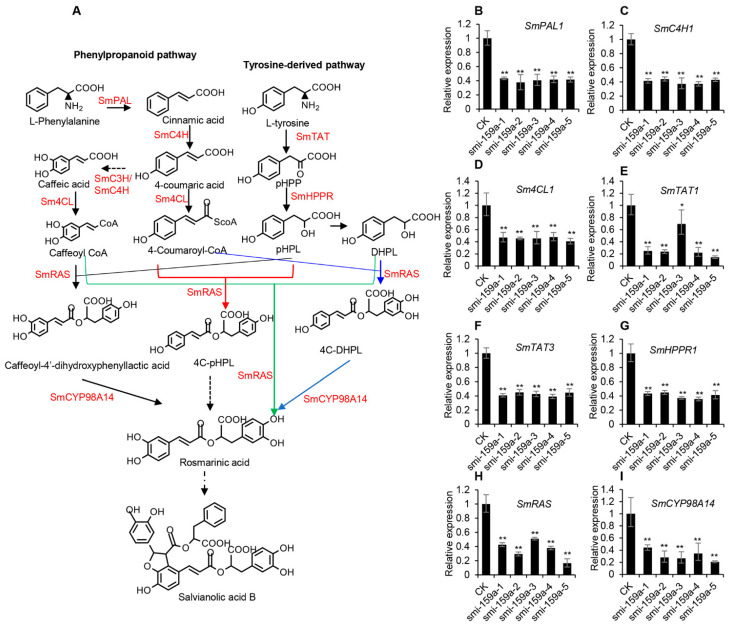
Relative expression levels of the key enzyme genes involved in RA and SalB biosynthesis. Dashed arrows represent multiple steps. (**A**) The biosynthetic pathways of phenolic acids based on the previous study [59,60]. PAL, phenylalanine ammonia lyase; C4H, cinnamate 4-hydroxylase; 4CL, 4-coumaroyl CoA ligase; TAT, tyrosine aminotransferase; HPPR, hydroxyl phenylpyruvate reductase; RAS, rosmarinic acid synthase; CYP, cytochrome P450 enzymes. (**B**–**I**) Relative gene expression levels in hairy roots of the control and Smi-miR159a overexpression transgenics. The expression levels were analyzed by qRT-PCR. *SmUBQ10* was used as a control. Expression levels in the control (CK) were set to 1. Error bars represent standard deviation of three biological replicates (** *p* < 0.01; * *p* < 0.05).

Further examining the expression of enzyme genes involved in the RA synthesis pathway using the qRT-PCR method showed that *SmPAL1*, *SmC4H1*, *Sm4CL1*, *SmTAT1*, *SmTAT3, SmHPPR1*, *SmRAS*, and *SmCYP98A14* were significantly decreased in the smi-miR159a-overexpressed hairy roots compared with the control [58] (Figure 7). The results suggested that smi-miR159a regulated RA and SalB biosynthesis through down-regulation of various enzyme genes.

## 3. Materials and Methods

### 3.1. Plant Materials and Sample Collection

*S. miltiorrhiza* (line 99-3) plants with whole-genome sequences available were propagated through root segments and grown in a field nursery located at the Institute of Medicinal Plant Development [40]. Two-year-old mature roots, young roots, stems, mature leaves, young leaves, and flowers were collected and immediately frozen in liquid nitrogen until use. 

### 3.2. sRNA and Degradome Sequencing

Total RNA was extracted from plant tissues using Trizol Reagent (Invitrogen, Carlsbad, CA, USA). Approximately 3 μg of total RNA from each tissue was used for sRNA library preparation. Six sRNA libraries, including Sm_MR, Sm_YR, Sm_S, Sm_ML, Sm_YL, and Sm_F, were constructed as described previously [61]. The libraries were sequenced on Illumina HiSeq2500 at LC-BIO (Hangzhou, China). A degradome library was constructed using pooled RNAs with equal amounts of RNA from each of the aforementioned six tissues. Approximately 20 μg of total RNA was used for degradome library preparation [62]. Single-end sequencing (50 bp) was performed on Illumina HiSeq2500 at LC-BIO (Hangzhou, China).

### 3.3. Data Processing and Computational Identification of miRNAs

After high-throughput sequencing, adaptor sequences, junk reads or/and reads with length < 18 nt or >25 nt were removed. Sequences with lengths of 18–25 nt were mapped to the mRNA database, Rfam (version 11.0) database (http://rfam.janelia.org/ accessed on 25 July 2018), and Repbase through BLAST search to remove mRNA, tRNAs, rRNAs, snRNAs, snoRNAs, and repeats. The remaining sequences were further BLAST-analyzed against the *S. miltiorrhiza* (99-3 line) genome assembly [31] to identify candidate precursors. Briefly, the reads were first mapped to the genome assembly and predicted for hairpin precursors using the psRobot software package v1.2 [49] with the default parameters, including minimal unique sRNA clone counts (2), maximum number of unique sRNA mapping locations in reference genome (20), minimal number of nucleotides between adjacent unique sRNA clusters (200), maximum length limitation of unique sRNA clusters selected to predict stem-loop precursors (300), count of unique sRNA cluster (≥10), highest expressed unique sRNA in one cluster (≥10), minimal number of mismatches in supposed miRNA mature region (1), and maximal number of mismatches in supposed miRNA mature region (5). The resulting hairpin structures were then manually checked using the following criteria: (1) a single-stranded, hairpin precursor with a free energy (dG) value less than 30, (2) minimal folding free energy index (MFEI) of at least 0.85 calculated as previously described [63], (3) no more than four mismatched miRNA bases as suggested by Meyers [64], (4) two or fewer asymmetric bulges within the miRNA/miRNA* duplex, (5) at least two reads for miRNAs, (6) the clone of miRNA* sequences being the most desirable, and (7) the miRNA/miRNA* duplex having a 2 nt 3′ overhang on each side. Finally, the verified miRNAs were mapped to the miRBase 22.0 for classification of known and novel miRNAs.

### 3.4. Prediction of miRNAs Targets and Degradome Analysis

miRNA targets were predicted using the online psRNATarget program [53]. The parameters used were as follows: a maximum expectation of 3.0, selection of the top 200 target genes for each small RNA, a length of 20 for complementarity scoring (hspsize), and a maximum energy of 25 allowed for target site unpairing (UPE).

Degradome data were analyzed using the CleaveLand 4.0 program [54]. Degradome reads were aligned to the predicted target genes. Fragment abundance of degradome reads and the category for each position in every gene were calculated. Subsequently, the potential targeting information and the degradome read alignments were merged to obtain a list of valid targets for each miRNA and generate the list with target cut site, category, and abundance. 

### 3.5. Experimental Verification of miRNA-Directed Target Cleavage

The cleavage sites were mapped with the modified 5′ RLM-RACE method using the SMARTTM RACE cDNA amplification kit (TaKaRa Bio, Otsu, Japan) as described before [63]. Briefly, nesting PCR amplification was carried out using the nesting primers listed in Table S9. A touchdown PCR program was applied as follows: 94 °C for 5 min, 5 cycles of 94 °C for 30 s and 72 °C for 3 min, 5 cycles of 94 °C for 30 s, 70 °C for 30 s and 72 °C for 3 min, and 25 cycles of 94 °C for 30 s, 56 °C for 30 s and 72 °C for 3 min, followed by extension at 72 °C for 10 min. The nested PCR amplification was performed using the nested primers listed in Table S9. The PCR program consisted of pre-denaturation at 94 °C for 5 min, followed by 25 cycles of 94 °C for 30 s, 56 °C for 30 s and 72 °C for 1 min, and final extension at 72 °C for 10 min. cDNA fragments were gel-purified and then cloned into pTOPO-TA vector (Aidlab, Beijing, China), followed by sequencing.

### 3.6. Quantitative Real-Time Reverse Transcription-PCR (qRT-PCR)

RNA used for qRT-PCR was from the same tissues for sRNA sequencing. For analysis of target gene expression, 1 μg RNA was reverse-transcribed into cDNA using the PrimeScript RT reagent kit (Takara, Shiga, Japan). Gene-specific primers were designed using Primer Premier 5 (Table S9). The expected length of amplicons ranged from 80 bp to 200 bp. *SmUBQ10* was utilized as an internal control. The expression of miRNAs was analyzed using the poly(A) adaptor RT-PCR method [65]. Primers used for the selected miRNAs were designed according to the recommended guidelines [65]. The 5.8S rRNA was chosen as a reference. Gene expression data from three biological replicates were normalized. ANOVA (analysis of variance) was performed using SPSS (Version 19.0, IBM, Chicago, IL, USA). A significance level of *p* < 0.05 was considered as statistically significant, and a significance level of *p* < 0.01 was considered as highly significant.

### 3.7. Plasmid Construction and Transformation

To overexpress smi-miR159a, an 876 bp sequence containing *smi-MIR159a* precursor was PCR-amplified from genomic DNA using gene-specific primers (Table S9). Through homologous recombination, the amplicons were cloned into pCAMBIA1391, in which overexpression of Smi-miR159a was driven by the double CaMV35S promoter. The resulting constructs were transferred into the *Agrobacterium* strain ACCC10060. Genetic transformation of *S. miltiorrhiza* was carried out using the *A. rhizogenes*-mediated transformation method [42]. Briefly, leaf explants of *S. miltiorrhiza* were inoculated with Smi-miR159a precursor-containing agrobacteria and then co-cultivated on 1/2 MS solid media in the dark for 48–72 h. Hairy roots were induced on a 1/2 MS solid medium supplemented with 200 mg L^−1^ of timentin and 30 mg L^−1^ of hygromycin, and then on a 1/2 MS solid medium with reduced timentin (150 mg L^−1^, 100 mg L^−1^, and 50 mg L^−1^) every 2 weeks. The resulting hairy roots with lengths of about 3–4 cm were then transferred to 100 mL of B5 liquid medium in 250 mL flasks and cultivated at 25 °C in the dark with shaking at 150 rpm. Hairy roots generated from leaf discs inoculated with empty vector-containing *Agrobacteria* were used as the control.

### 3.8. UPLC Determination of RA and SalB

Sixty-day-old hairy roots were harvested and dried at 50 °C. To extract phenolic acids, dried hairy root powder was weighed, dissolved in 10 mL of 80% methanol (Thermo-Fisher Scientific, Waltham, MA, USA), and sonicated (37 kHz, 100% power) for 60 min using a bath sonicator. The supernatant was collected, concentrated under nitrogen, and then dissolved in 2 mL of methanol. The extractions were filtered using 0.22 µm Millipore Express PES membrane filters and analyzed using UPLC (Waters, Milford, MA, USA). Briefly, the filtrate (2.0 µL) was injected into an ACQUITY UPLC BEH C18 column (1.7 μm, 100 mm × 2.1 mm) at a flow rate of 0.3 mL min^−1^ and constant temperature of 25 °C and analyzed using UPLC-UV under the following conditions. The mobile phase A was 0.1% (*v*/*v*) formic acid (MREDA, Beijing, China)–methanol (Thermo-Fisher Scientific, MA, USA). The mobile phase B was 0.1% (*v*/*v*) formic acid (MREDA, Beijing, China) in water. The mobile phases changed with the following gradients: 0–6 min, 5% A; 6–8 min, 5–20% A; 8–14 min, 20–21% A; 14–18 min, 21–95% A. After the last gradient step, the column was equilibrated and washed with 5% A for 2 min. Chromatograms were recorded at 280 nm. The chemical constituents in the extract were characterized based on comparisons of retention times to RA and SalB standards (Push Bio-technology, Chengdu, China). All measurements were analyzed in triplicates. 

### 3.9. UPLC Determination of Tanshinones

Extraction and analysis of tanshinones were carried out as described by Pan et al. [42] with modifications. Briefly, hairy roots were dried at 50 °C and then ground into powder. The powder (0.1 g) was extracted three times with methanol (1.0 mL) under ultra-sonication (37 kHz, 100% power) for 30 min and centrifugation at 10,000 rpm for 10 min. Supernatant was filtered through a 0.22 µm Millipore filter and then used for UPLC analysis. Chromatography analysis was performed on a Waters Acquity UPLC (Waters, Milford, MA, USA) with an ACQUITY UPLC BEH C18 column (1.7 μm, 100 mm × 2.1 mm). Sample injection volume was set at 2 µL and detection wavelength was 270 nm. Separation was achieved by elution using a gradient with solvent A (water with 0.1% (*v*/*v*) formic acid) and solvent B (100% acetonitrile). The gradient elution was performed as follows: 0–5 min, 40% B; 5–25 min, 40–60% B. The flow rate was set at 0.3 mL·min^−1^. Chemical constituents in the extract were characterized based on the comparisons of retention times to the standards purchased from Push Bio-technology (Chengdu, China). 

### 3.10. Expression Analysis of Key Enzyme Genes

Total RNA from sixty-day-old hairy roots of the control line and *smi-MIR159a* transgenic lines was extracted using the Quick RNA Isolation Kit (Huayueyang, Beijing, China). cDNA was synthesized from 1 μg of total RNA using the M5 Super plus qPCR RT kit with gDNA remover (Mei5bio, Beijing, China) as per the supplier’s instructions. Gene expression for three replicates was determined using qRT-PCR with the SYBR Green qPCR Mix (Aidlab, Beijing, China). *SmUBQ10* was utilized as an internal control. The primers used are listed in Table S9. The following standard thermal profile was used for qRT-PCR: 95 °C for 3 min; 40 cycles of 95 °C for 10 s; 60 °C for 15 s; and 72 °C for 20 s.

### 3.11. Statistical Analysis

Statistical analysis was performed using SPSS 19.0 statistical software (IBM, USA). Data were expressed as mean ± SD. Comparisons between two groups were assessed by Student’s *t*-test. Comparisons among multiple groups were assessed by one-way analysis. *p* < 0.05 was used as the criterion for statistical significance.

## 4. Conclusions

*S. miltiorrhiza* is a well-known medicinal herb used for treating cardiovascular and cerebrovascular diseases. Its pharmacologically active compounds mainly consist of phenolic acids and tanshinones. In this study, we constructed six sRNA libraries for different tissues of *S. miltiorrhiza*, including mature roots, young roots, stems, mature leaves, young leaves, and flowers. A total of 137 known and 49 novel miRNAs were identified (Table S2). Most known miRNA families were conserved among plant species, indicating their roles in preserving key functions. The novel miRNAs likely emerged recently and could be involved in species-specific aspects of plants. A functional analysis of smi-miR159a clearly showed its regulatory role in *SmMYB62*, *SmMYB78,* and *SmMYB80*, which further affected phenolic acid biosynthesis through down-regulating the expression of *SmPAL1*, *SmC4H1*, *Sm4CL1*, *SmTAT1*, *SmTAT3*, *SmRAS1,* and *SmCYP98A14*. However, the underlying regulatory mechanism between SmMYBs and key enzyme genes remains to be investigated. In addition, candidate *S. miltiorrhiza* miRNAs identified in this study need to be further validated. The roles of these miRNAs, particularly those potentially to be involved in bioactive compound biosynthesis, need to be analyzed through genetic manipulation. Our results reveal new functions of miR159 and provide novel insights into the regulation of phenolic compound biosynthesis in plants. It is useful for the construction of miRNA-associated regulatory networks and improvement of bioactive compound contents in *S. miltiorrhiza*.

## Data Availability

The data are available in the article and its Supplementary Data.

## Supplementary Materials

The following supporting information can be downloaded at: https://www.mdpi.com/article/10.3390/ijms25105148/s1.
