# Supplementary material for: Genome-Wide Identification and Functional Analysis of Salvia miltiorrhiza MicroRNAs Reveal the Negative Regulatory Role of Smi-miR159a in Phenolic Acid Biosynthesis
Source: Int J Mol Sci. 2024 May 9;25(10):5148. doi: 10.3390/ijms25105148 (PMC11121111; doi:10.3390/ijms25105148)
Supplement: Supplementary file 1 [file ijms-25-05148-s001.zip › Supplemental Figure S1.pdf]

smi-MIRN1 (dG=-52.7)

```
                                        AG   AAAU     G      AAAGGUUUACACGA
5'-UUCACGUGAUAAUGCAAAUUGAACAAUUUUAUGUGAU  UGC   UCACGC AAAAUC              \
   ||| |||||| ||||||||||||||||||  ||  ||  |||   |||||| ||||||
3'-AGGAGCGCUAAUACGUUUAACUUGUUAAAGCACGUUA  ACG   AGUGUG UUUUAG              U
                                        A-   AU--     -      CAUAUUAAUAACGC
```

smi-MIRN2 (dG=-120.6)

```
                       -                  UUU   CUUG         UUG
5'-GUUGCUUUAGAAAUUGUGUG GCUUAAUGAAUCGUGACA   UAUU   UUAUUAUCU   A
   |||||||  ||||||||||| ||||||||||||||  ||   ||||   |||||| ||
3'-CAACGAACCCUUUGACACAC CGGAUUACUUAGUAAGGU   AUAA   AGUAAUUGA   A
                       U                  UU-   UAA-         UGU
```

smi-MIRN3a (dG=-57.3)

```
           A---                 C             AAA-   UCG
5'-CUCUUGUC    CGCAACUACAUCGCCGG GAUCACCAGAAUC    GUG   \
   ||||||||    ||||||||||||| ||| |||||||||||||    |||
3'-GAGAACGG    GUGUUGGUGUAGUAGCC UUAGUGGUCUUAG    CAC   C
           GCGA                 -             AACG   UAC
```

smi-MIRN3b (dG=-47.9)

```
                    C             AA     CGC
5'-CGCAACUACAUCGCCGG GAUCACCAGAAUC  UGUGU   \
   ||||||||||||| ||| |||||||||||||  |||||
3'-GUGUUGGUGUAGUAGCC UUAGUGGUCUUAG  ACGCA   C
                    -             A-     CUA
```

smi-MIRN4 (dG=-49.3)

```
           --                  G G           -  UGU
5'-CAACAUGU  UUGUUUGUGGUGCAAUGG C AACGCCGAGGU GG   \
   ||||||||  ||||  |||||||||||| | ||||||| ||| ||
3'-GUUGUAUA  AACACUCACCACGUUACU G UUGCGGCCCCA CC   C
           CU                  - -           A  UGA
```

smi-MIRN5a (dG=-56.5)

```
                                AAACAAAGCU--          --            G
5'-UGAUUUGCUCGGGCUUUCAGAAUGUAAUAA           AUGAAUGUGU  UUAUUGUUACU U
    ||||| ||||||||||||||||||||||           |||| ||||  ||||||||||  |
3'-ACUAAUCGAGUUCGAGAGUCUUACAUUGUU           UAUUCUCACA  AGUAGCGGUGG A
                                ACAGCAGCUGUU          GU            U
```

smi-MIRN5b (dG=-67.2)

```
          AGAAGGAA                                    UAACAGGGCCGAAGAU        U
5'-GAGGAGG        GAGUGUUUGAUUAGCUCGAGCUUUCAGAAUGUAACA                UGUCAUUG A
    |||||||         ||||| ||||||||||||||||||||||||||||                ||||||||
3'-CUCCUUC        CUUACCGACUAAUCGAGUUCGAGAGUCUUACAUUGU                AUAGUAGC C
          --------                                    U---------------        A
```

smi-MIRN6 (dG=-104.7)

```
                                                         U         AUCAACAUAUAUUA
5'-AUCACCUACCCAUCGUGGGCAGACAUAAUAUGUUCACCACUGAUAUGACAAUUUUAAUU GAAAAGAGA              \
    ||||||||||||||||||||||||||||  |||  |||||||||  ||||||||||||| |||  ||||
3'-UAGUGGAUGGGUGGCACCCGUCUGUAUUACACGUGUGGUGACUACACUGUUAGAAUUAA UUUCAUUCU              C
                                                         -         AUUAAAAUUAAUCU
```

smi-MIRN7 (dG=-77.8)

```
                                       -------     AAAAC-       UCCC      U
5'-CCGAUGGUGAAGGUGGAGAUGAGAUAGUUGAGCAAA       ACACC      UUGAAUCA    UCGAAAU \
    ||||||||| |||||||||||||||||||||||||       |||||      |||||||||   |||||||
3'-GGCUACCACUGCCACUUCUACUCUAUCAACUCGUUU       UGUGG      AGCUUGGU    AGUUUUG U
                                       UAUUGGC     CUUAUU       UA--      C
```

smi-MIRN8 (dG=-108.2)

```
            CAC           UAAG---                  UA      UUA        AUGAG                      -                AAUUUU--       UUG
GCU
5'-GCACUCUCGU    GGGUCUUUGU          AUAAGUUGGUUGAAGU  AAAAAU    UUUAAAA       GGUGCUUUAGAAAUUGUGUG GCUUAAUGAAUCAU             UUGUUC
UUAUUGCUAGCGU   \
    ||||||||||    |||  ||||||          ||||||| ||||||| ||||||    |||||||       ||||||| |||||||||||| ||||||||||||||             ||||||    ||||||   ||
|
3'-UGUGAGAGCA    UCCUGAAAUA          UAUUCAAACAACUUCG  UUUUUA    AAAUUUU       CUACGAACCCUUUGACACGC CGGGUUACUUAGUA             AAUAAG
AGUAACCUUUAUA   U
            UUU           UAUUUAA                  --      UC-        AUAA-                      U                AGUUUUAU       UA-
AAA
```

smi-MIRN9 (dG=-75.6)

```
                         CAGA                  -          AAGA-      UAGGU
5'-CUAUACUUACGGGUGAUUCUGC    GUGGACCGACGGCCAUC  UGCACUGUGA    UUCGGAU     \
   ||||||||||||||||||||||    |||||  |||||| |||  ||||||||||    |||||||
3'-GAUGUGAGUGCCCAUUAAGACG    CACUUAACUGCUGUUAG  AUGUGACACU    AAGCCUG     C
                         CC--                  G          ACCAA      UAAAA
```

smi-MIRN10 (dG=-60.6)

```
                                               C     U       -    GA
5'-GAGUUUCAUUUUCUAUUCGAAUCGUUGGAUUGUUUUCAUGUAUA UUUGA AAAUUUU CGAU   \
   | ||||||||||||||||  |||||||||||||| ||||||||| ||  | ||||||| ||||
3'-CAUAGAGUAGAAGAUAAGGCUAGCAACCUGACAGCAGUGCAUAU AAGUU UUUAGAG GCUG   A
                                               -     -       U    GU
```

smi-MIRN11 (dG=-81.1)

```
                     U        U                               CG-------     UA
5'-GCAAGAGACUUCCAUGGA AAGUAGAG GGUGGAUUGCAGCCAAGGAUGACUUGCCGGC         CCCGG  \
   ||  |||| || |||||| |||||||| |||||  || ||||||||||  |||||||||         |||||
3'-CGGCCUCUCAGCGUACUU UUCGUCUC CCGCUUUACAUCGGUUUCUGUCGAACGGCCG         GGGCC  U
                     -        -                               CCUGGGAAA     AG
```

smi-MIRN12 (dG=-59.2)

```
                                    -    AACAA      CAUUGCCACCC
5'-GCAGACGGAGGACAAGGGGAAUUAGGGCUCAAG GGGA     AGAUAA           \
   |||||||  ||| |||||||||||||||||||| ||||     ||||||
3'-CGUCUGCUCCCUCUUCCCUUUAAUCUCGAGUUC UCCU     UCUAUU           A
                                    U    AUCUC      AUUUUCUUACU
```

smi-MIRN13 (dG=-121.7)

```
                                                        A-   G
5'-AAAGAUGUAGUCAUACUAGCCGGUCAGAGCAAUAUGAUCGGCGAAUGCCAUAU  CCUU \
   ||||||||  ||||||| ||||| ||||||||||||  ||| ||||||| |||  ||||
3'-UUUUUAUACAAGUAUGAACGGCCCGUCUCGUUAUACCGGCCUCUUACGGCAUA  GGAA G
                                                        AC   C
```

smi-MIRN14 (dG=-67.8)

```
     U                 -                     C  UA
5'-UG CCUCUCUCUUCUCUCGC UUGUCUCUUCCGAACCCUUCC GU  A
   || |||||||| |||||| | |||||||||||||||||||| ||  |
3'-AC GGAGAGAGUAGAGAGGG AACAGAGAAGGCUUGGGAAGG UA  U
     -                 G                     -  UU
```

smi-MIRN15 (dG=-76.1)

```
                    C              AC                  CAU  CUA         GUA
5'-UUUUUAACGAUAGGUAG UCUAUCAUCAUCGG  CACCGACGUGGUCAGAAU   GC   AAAGUUAGU   A
   |||||||| ||||||| |||||||||||||  |||||| ||||||||||   ||   |||||| ||
3'-AAAGGUUGUCAUCCAUC AGAUAGUAGUAGCU  GUGGCUACACCAGUUUUA   UG   UUUUAACCG   G
                    -              A-                  AUU  AAC         AGU
```

smi-MIRN16 (dG=-147.8)

```
                                                                         ----      A
5'-CCUCAAGUAUGCAGCUGCUUCACUAAGUGGAUUGCCUCAGAAGCUAUCACCGUGACAGUAGCAGAGUCUG    GCUAGU U
   || |||||||||||||||||||||||||||||||||||||||||||||||||||||||||||||||||    ||||||
3'-GGUGUUCAUACGUCGACGAAGUGAUUCACCUAACGGAGUCUUCGAUAGUGGCACUGUCAUCGUCUCAGAC    CGAUCA U
                                                                         UNGUA     A
```

smi-MIRN17 (dG=-42.8)

```
                                 UAUUUCU  UUUC-      GAUA
5'-UAUAUGUAUAUUUGAUCUUUGCCAUAUGUA       UC     AAAAUG    \
   ||||||||||||||||||||||||||||||       ||     ||||||
3'-AUAUAUAUAUGAACUAGAAACGGUGUAUAU       AG     UUUUAC    U
                                 UUUAUAU  CUCCA      GGUA
```

smi-MIRN18 (dG=-101.9)

```
              C                                     CACACU     - UC
5'-AGACCUCGGGC UUGGGCAUUAACCACCUUACUGCUAGGCCAACACACG      GGUGUG C  \
   ||||||  ||| ||||||||||||||| ||||| || ||||||||||      |||||| |
3'-UCUGGAAAUCG AAUCCGUAAUUGGUGAGAUGAGGACCCGGUUGUGUGC      UUACAC G  A
              -                                     AU----     U CU
```

smi-MIRN19 (dG=-47.4)

```
     AUC                            CCU      GAA    UUG
5'-GA   CCAUUAUCGGAAAAUCCAUGUAGCAUG   GAUCUGA   UGAAG   \
   ||   ||| ||||||||||||||||| |||||   |||||||   |||||
3'-CU   GGUCGUAGCCUUUUAGGUGUACCGUAC   UUAGACU   ACUUU   U
     GAA                            AGU      GC-    UGU
```

smi-MIRN20 (dG=-99.3)

```
                                      ----  AAUU-          AUG      CAUAU
5'- UUUGUUGCAAUGUCCGUCCGGAGAUACCUUCCAU    GC      UUUUGUUGCA   UCUAU      \
    ||||||||||||||||||||||||||||||||||    ||      |||  |||||   |||||
3'- AAACAACGUUACAGGCAGGCCUCUAUGGAAGGUA    CG      AAAGUAACGU   AGGUA      C
                                      AUUC  AAAGU          ---      CAUCA
```

smi-MIRN21 (dG=-68.0)

```
                                           CAA       A---           A      C
5'-UAAAUUAAGUGAGCUGGAAAAUUGAGAUAUCCCAUGUCAU    CAUGAC     UUGUUUUUUGU CUUUUU U
   |||||| |||||| ||||||||||||||||||||||||||    ||||||     ||||||| ||| ||||||
3'-AUUUAAAUUACUCCACCUUUUAACUCUAUAGGGUACAGUG    GUAUUG     AGCAAAACACA GAAAAA U

                                           ---       AUAC           -      A
```

smi-MIRN22 (dG=-53.6)

```
          ACC                  -            ACAUACA                   AU
5'-UGGUUGC   GUGCAAUUGGUUUCCGGA UGACACUAUUUCA       AAUGACAUUUGAAGAUCUC  \
   |||| |    ||||||| |||||||||| ||||| |||||||       ||||| ||||||| || ||
3'-AUCAGUG   CACGUUGUCUAAAGGUCU ACUGUAAUGAAGU       UUACUAUGAACUUGUAAAG  U
          ---                  U            AAGCACA                   AC
```

smi-MIRN23 (dG=-97.4)

```
                                        GU        AAUAUU      CACCCAU         --        A
5'-UGCUUCGUACUGCUCCUCACUUAUAGCCACAUCUGCU  GUGUGUCAA      CACCUG       GAGGUCUAU   GAGUAUC G
   |||||||||||||||||||||||||||||||| ||||  |||||||||      ||||||       ||||| |||   ||| |||
3'-ACGAAGCAUGACGAGGAGUGAAUAUCGGUGUAAACGA  UACACAGUU      GUGGAC       UUCCAAAUA   CUCUUAG A
                                        --        AAAU--      CU-----         AU        A
```

smi-MIRN24 (dG=-65.4)

```
            ACC       CUACACUAAGU        ACG                              AA
5'-CAAGCAUGU   UGUACUA           CACUAACCC   GAUUUUGCGACUGGCGACAAUUGGAUGUA  A
   || ||||||   |||||||           |||||||||   ||||||||||| |||| || |||||| ||
3'-GUCCGUACG   ACAUGAU           GUGAUUGGG   CUAAAACGCUGUCCGUCGUCAACCUGGGU  C
            GAU       -----------        AAA                              GA
```

smi-MIRN25 (dG=-72.3)

```
                                                 GCU        C-    AUAA-                AUUC
5’-UUUUCCGAUGCCGAAAAACACAAUUGGCAACCAGAUUUCAUGUGUG   UUUCUUCU  AGUA     AUUUAAUGAUGUGGAA    \
   ||||||| |||||| ||| ||| ||||||||| ||||||||| |||   ||||| ||  ||||     |||| |||||||||||
3’-AAAAGGUCACGGUUCUUUAUGUCAACCGUUGGCCUAAAGUACUCAC   AAGGACGA  UUAU     UAAACUACUACACCUU    A
                                                 AGU        AC    AGACC                AUAG
```

smi-MIRN26 (dG=-64.6)

```
              AUGGA     UAC                             AACUUUUC
5’-CAAUCCAUUGA     UUUAC   ACGUGGCAGUUGGAGCCAUCAUUUUGCAC        \
   |||||||||||     |||||   |||||||||||||| ||||||||||||||
3’-GUUGGGUAACU     AAGUG   UGCACCGUUAACCUGGGUAGUAAAACGUG        C
              A----     CUA                             CCUUUUAC
```

smi-MIRN27 (dG=-81.2)

```
                           -              AAUAUAACACAUACAAGAUCGAAAA
5’-AAAUACAUGAACAAGAGAUGGUCG AUCGUGAACAAUUA                         U
   ||||| |||||||||||||||||| |||||||| |||||
3’-UUUAUAUACUUGUUCUCUACCAGU UAGCACUUAUUAAU                         C
                           A              AUUAUGUACUUGUUAGUGUAAUCAA
```

smi-MIRN28 (dG=-78.8)

```
               CAC                                         AUAUA
5’-UGCUAACGUUUG   GGUGCAGAUCACGCACAUACUCUUAUAGGAUACUUGUAAGU     \
   ||| ||||| ||   |||||||||||||||||||||||||||||||||||||||||
3’-ACGUUUGUAUAC   CCAUGUCUAGUGUGUGUAUGAGAAUAUCCUAUGAACAUUCA     U
               AUC                                         ACAUU
```

smi-MIRN29 (dG=-64.3)

```
        UGA--                                       UAU
5’-CUCUG     UACUCCCUCCAUCCCGGUUAUAACGUCCCAAAGUUAUAG   \
   |||||     |||||||||| |||||||||||| ||||||||  |||||
3’-GAGAU     AUGAGGGAGGCAGGGCCAAUAUUACAGGGUUUUGAUAUC   U
        UUAGG                                       CUG
```

smi-MIRN30 (dG=-61.5)

```
      CU                                             AAU
5’-AUA  AACUACUACUCCCUCCGUCCCACUUCAAAUAUCUCAAAACUAUAG   \
   |||  ||||| ||||||||||||||||||| |||| |||||||| |||||
3’-UAU  UUGAUUGUGAGGGAGGCAGGGUGAAAUUUACAGAGUUUUAAUAUC   U
      U-                                             CUG
```

smi-MIRN31 (dG=-114.9)

```
      C                         -                             AGAA---    AU
5’-UAU GCUAAAACACGAUCGGUCCGGCCGG UGAAGUGGCCGGAUAAAUCGAUCCGGCCG       CAUU  \
   ||| |||||||||||||||||||||| || |||||||||||||||||||||||||||||       ||||
3’-AUA CGAUUUUGUGCUAGCCAGGCCGACC ACUUCACCGGCCUAUUUAGCUAGGCCGGC       GUAA  U
      -                         A                             AUACAAA    AU
```

smi-MIRN32 (dG=-119.8)

```
             UUCA-                         C   C-      CAA     AA
5’-GCGGCUACUG     CGGCUAUGAAUUUGUGGCGGCUAAU UAC  CAUUUG   CAAAA  \
   ||||||||||     |||||||||| || ||||||||||| |||  ||||||   || ||
3’-CGCCGAUGAC     GCCGAUACUUUAAGACCGCCGAUUA AUG  GUAAAC   GUAUU  A
             UCAUC                         -   CA      AAA     AU
```

smi-MIRN33 (dG=-76.1)

```
                                                                        U
5’-UGUGAAAUCGGUUUUAUAGUGAAACCGACUUCACAAUGACACUGCUUCAAUAUACAAAUGACAUUUUAU A
   ||||||  ||||||||| |||||| || |||||||||||||||||||||||||| |||| ||||||||| |
3’-ACAUUUGGGCUAAAAUACUACUUUAGCCGAAGUGUUACUGUGACGAAGUUGUAUAUUUAAUGUGAAAUA U
                                                                        U
```

smi-MIRN34 (dG=-66.1)

```
         UU---                                           AA
5’-AGUCUC     GUCAAGAUCACUCAACUGAAUCGUAGCAUCACUCGUAUACCAA  \
   || |||     ||||||||||||||| ||||| |||||||||||||||||||||
3’-UCCGAG     CAGUUCUAGUGAGUUUGCUUAACAUCGUAGUGGGUAUAUGGUU  C
         UUCAU                                           GU
```

smi-MIRN35(dG=-98.9)

```
                                  AA                               AUUGUAAUACAUAAAAAAACA
5'-GGUGAAAACUAGAUCCUAUGAACAAACACAUA  AUACGUGAACAAGAGAUAAUCAAGUUGUGAACA                   U
   ||||||||||||||||||||||||||||||||  |||||||||||||||||| ||||||||||||||
3'-CCACUUUUGAUUUAGGAUACUUGUUUGUGUAU  UAUGCACUUGUUCUCUAUCAGUUUAGCACUUGU                   G
                                  G-                               CCUAAGCUGGUAGAGAACAA
```

smi-MIRN36 (dG=-132.1)

```
                                         AUGUUA                  ACGAAUUUU  G
5'-GAGUGCCUUUUUCGAUGGUCUUGCCUGUCUUUCUCUGU      UAUAUUGUUGAUUG         UG A
   ||||||||||||||||||||||||||||||||||||||       ||||||||||| ||         ||
3'-CUCACGGAAGAGGUUACCAGAACGGACAGAAAGAGACA      AUAUAGCAACUCAC         AC A
                                         C-----                  ---------  A
```

smi-MIRN37 (dG=-57.0)

```
          -          -  AA                                AACCUA
5'-CAAAAUU AAGAUUCUGG CA  AUCGAAACUGGCGUGACAUUGGAAUUUUUAGA      G
   ||||||| |||||||||| ||  ||||||||||| |||| |||||||| ||||||      |
3'-GUUUUAA UUCUAAGGCC GU  UAGCUUUGACCACACUUUAACCUUACAAAUCU      C
          U          A  G-                                GCAACC
```

smi-MIRN38 (dG=-50.7)

```
      A                                UUUUGGUCGUCCCAUUUUUAUAC      CUUGCUC
5'-UCC UCCGUCGCACUUCACUUGGCUUAGUUUUUUUU                       UACAAU       U
   ||| |||||| |||||||||||  ||||||||||||                       ||||||
3'-AGG AGGCAGGGUGGAGUGAACUUGAUCAAGAAAAG                       GUGUUA       U
      -                                CC---------------------      AUAAUUU
```

smi-MIRN39 (dG=-80.4)

```
                     -   A                                   GGCCA            AA
5'-GAUUUUCAAUAGUAGGUA CC CAUCAUCAUCGGACCACCGGUGUAGUCGGAAUCG     AAAAAUUAGUA   A
   |||||||||||||||||| || |||  |||||||||| |||||  ||||||||||     ||||||| |||   |
3'-CUAAAAGUUGUUAUCCAU GG GUACCAGUAGCCUGGUAGCCACUCCAGCCUUAGU     UUUUUAACCGU   U
                     C   -                                   GCAAA            AG
```

smi-MIRN40 (dG=-74.8)

```
                   -         -              -----   GGG          GAAAAU
5'-UCUUGAAUAUCACCGG GAAUUGCAC GGGAGAGGAGACGUC     GU   GCCGAUUUUC      \
   || ||||||||||||| |||||||| |||||| |||||||     ||   ||||||||||
3'-AGUAUUUAUAGUGGCC UUUAACGUG CCCUCUGCUUUGCAG     CG   UGGCUAAAAG      U
                   U         C              GGAGG   GG-          GGCGGU
```

smi-MIRN41 (dG=-140.5)

```
         C            -                                 GG
5'-UUUUUC ACAUAUCCUACU GGCCCUUACUCAUUCCACAUUUACACUACAAAGAG  A
   |||||| |||||||||||| ||||||||||||||||||||||||||||||||||   |
3'-AAAAAG UGUAUAGGAUGA CCGGGAAUGAGUAAGGUGUAAAUGUGAUGUUUCUU  U
         -            A                                 AA
```

smi-MIRN42 (dG=-80.2)

```
                         U-                                        UGA
5'-CUCCCGUGAAACCGGUUUUUUUU  AAAACCACCUUUACAAUGACACUACUUCAACAUACAAA   \
   |||| ||||||| |||||||||  |||||||| ||||||||||||||||||||||||||||
3'-GAGGACACUUUGUCUAAAAAAA  UUUUGGUGUAAAUGUUACUGUGAUGAAGUUGUAUGUUU   U
                         UU                                        UUA
```

smi-MIRN43 (dG=-79.5)

```
                                          CAUUUUUUACAAAAGAGAAUUUCACCC                 UC
5'-AAAUUUCUCCCUUCAAAUCCCUCCUUUCUUUUCCCU                           UUUAUCAUUCCUCUUUG  A
   ||||||||||||||||||||||||||||||||||||                           ||||| |||||||||||
3'-UUUAAAGAGGGAAGUUUAGGGAGGAAAGGAAAGGGA                           AAAUAAUAGGGAGGAAC  C
                                          ------------------------AUC                 CU
```

smi-MIRN44 (dG=-82.8)

```
                                                   AG---------      GC
5'-UUGUUAUUAUCUUGCAGGGCAUGGCAUUUUAUGAUUAAUUCGUGGUG           GAGGGG  U
   ||||| ||||||||||||||||||||||||||||||||||||| ||           ||||||
3'-AACAACAAUAGAACGUCCCGUACCGUAAAAUACUAAUUAAGCACAAC           CUUCUC  U
                                                   AAUGUCGACGA      GA
```

smi-MIRN45 (dG=-89.5)

```
                                                 -        UUUA
5’-GUGGGCAAGAAUAAUGUGCCCACCAUUGAUGUGGCAAUGUUAAUUA GAGUAAGA    \
    ||||||||||||||||||||||||||||||||||||||||||||| |||  |||
3’-CACCCGUUCUUAUUGCACGGGUGGUGACUACACCGUUACAAUUAAU CUUUCUCU    U
                                                 C        UAAU
```

Figure S1. Predicted hairpin structures of *S. miltiorrhiza* novel miRNA precursors. Mature miRNA sequences are indicated in red and miRNA* sequences are indicated in blue.
